# Supplementary material for: Coherently driven microcavity-polaritons and the question of superfluidity
Source: Nat Commun. 2018 Oct 3;9:4062. doi: 10.1038/s41467-018-06436-2 (PMC6170389; doi:10.1038/s41467-018-06436-2)
Supplement: Supplementary file 1 — Supplementary Information [file 41467_2018_6436_MOESM1_ESM.pdf]

Supplementary information
Coherently driven microcavity-polaritons and the question of superfluidity

Juggins et al.

Supplementary Note 1 - Response Function Coefficients

Using the shorthands,

$$J(\mathbf{q}) \equiv \omega_p - \epsilon_{\mathbf{q}} + \mathrm{i}\kappa - 2V\left|\psi_0\right|^2, \tag{1}$$

$$K(\mathbf{q}) \equiv -\frac{2}{\det[(D^{\mathrm{R}})^{-1}(\omega=0,\mathbf{q})]} = -\frac{2}{J(\mathbf{q})J^*(-\mathbf{q}) - V^2\left|\psi_0\right|^4}, \tag{2}$$

$$L_1 \equiv J(\mathbf{0}) + J^*(\mathbf{0}) + 2V\left|\psi_0\right|^2 = 2\Delta - 2V\left|\psi_0\right|^2, \tag{3}$$

$$L_2 \equiv J(\mathbf{0}) + V\left|\psi_0\right|^2 = \Delta + \mathrm{i}\kappa - V\left|\psi_0\right|^2, \tag{4}$$

where $\epsilon_{\mathbf{q}} = (\mathbf{q}+\mathbf{k}_{\mathrm{p}})^2/2m^*$, the coefficients in Eqs. (7-10) are given by:

$$c^{\mathrm{mf}}_{+,+}(\mathbf{q}) = \frac{1}{2}\left|\psi_0\right|^2 K(\mathbf{q})J^*(-\mathbf{q}), \tag{5}$$

$$c^{\mathrm{mf}}_{+,-}(\mathbf{q}) = c^{(0)}_{-,+}(\mathbf{q}) = \frac{1}{2}\left|\psi_0\right|^2 K(\mathbf{q})V\left|\psi_0\right|^2, \tag{6}$$

$$c^{\mathrm{mf}}_{-,-}(\mathbf{q}) = \frac{1}{2}\left|\psi_0\right|^2 K(\mathbf{q})J(\mathbf{q}), \tag{7}$$

$$\mathcal{C}^{\mathcal{A}}_{+}(\mathbf{q}) = -VK(\mathbf{q})\begin{pmatrix}\left|\psi_0\right|^2[J^*(-\mathbf{q})+V\left|\psi_0\right|^2] & \psi_0^2 J^*(-\mathbf{q}) \\ V\bar{\psi}_0^3\psi_0 & \left|\psi_0\right|^2[J^*(-\mathbf{q})+V\left|\psi_0\right|^2]\end{pmatrix}, \tag{8}$$

$$\mathcal{C}^{\mathcal{A}}_{-}(\mathbf{q}) = -VK(\mathbf{q})\begin{pmatrix}\left|\psi_0\right|^2[J(\mathbf{q})+V\left|\psi_0\right|^2] & V\bar{\psi}_0\psi_0^3 \\ \bar{\psi}_0^2 J(\mathbf{q}) & \left|\psi_0\right|^2[J(\mathbf{q})+V\left|\psi_0\right|^2]\end{pmatrix}, \tag{9}$$

$$\mathcal{C}^{\mathcal{B}}_{+}(\mathbf{q}) = -VK(\mathbf{q})\begin{pmatrix}\left|\psi_0\right|^2[J^*(-\mathbf{q})+V\left|\psi_0\right|^2] & V\bar{\psi}_0\psi_0^3 \\ \bar{\psi}_0^2 J^*(-\mathbf{q}) & \left|\psi_0\right|^2[J^*(-\mathbf{q})+V\left|\psi_0\right|^2]\end{pmatrix}, \tag{10}$$

$$\mathcal{C}^{\mathcal{B}}_{-}(\mathbf{q}) = -VK(\mathbf{q})\begin{pmatrix}\left|\psi_0\right|^2[J(\mathbf{q})+V\left|\psi_0\right|^2] & \psi_0^2 J(\mathbf{q}) \\ V\bar{\psi}_0^3\psi_0 & \left|\psi_0\right|^2[J(\mathbf{q})+V\left|\psi_0\right|^2]\end{pmatrix}, \tag{11}$$

$$\mathcal{C}^{\mathcal{M}}_{+,+}(\mathbf{q}) = -\frac{1}{2}VK(\mathbf{q})\begin{pmatrix}\begin{matrix}\left|\psi_0\right|^2\big(K(\mathbf{q})[1-K(\mathbf{0})V\left|\psi_0\right|^2 L_1] \\ \times[J^*(-\mathbf{q})^2+V^2\left|\psi_0\right|^4] \\ -K(\mathbf{0})J^*(-\mathbf{q})L_1[K(\mathbf{q})V^2\left|\psi_0\right|^4-1]\big)\end{matrix} & \begin{matrix}\psi_0^2\big(-K(\mathbf{0})K(\mathbf{q})V\left|\psi_0\right|^2 L_2 \\ \times[J^*(-\mathbf{q})^2+V^2\left|\psi_0\right|^4] \\ +J^*(-\mathbf{q})(K(\mathbf{q})V\left|\psi_0\right|^2 \\ -K(\mathbf{0})L_2[K(\mathbf{q})V^2\left|\psi_0\right|^4-1])\big)\end{matrix} \\ \begin{matrix}\bar{\psi}_0^2\big(-K(\mathbf{0})K(\mathbf{q})V\left|\psi_0\right|^2 L_2^* \\ \times[J^*(-\mathbf{q})^2+V^2\left|\psi_0\right|^4] \\ +J^*(-\mathbf{q})(K(\mathbf{q})V\left|\psi_0\right|^2 \\ -K(\mathbf{0})L_2^*[K(\mathbf{q})V^2\left|\psi_0\right|^4-1])\big)\end{matrix} & \begin{matrix}\left|\psi_0\right|^2\big(K(\mathbf{q})[1-K(\mathbf{0})V\left|\psi_0\right|^2 L_1] \\ \times[J^*(-\mathbf{q})^2+V^2\left|\psi_0\right|^4] \\ -K(\mathbf{0})J^*(-\mathbf{q})L_1[K(\mathbf{q})V^2\left|\psi_0\right|^4-1]\big)\end{matrix}\end{pmatrix}, \tag{12}$$

$$\mathcal{C}^{\mathcal{M}}_{+,-}(\mathbf{q}) = -\frac{1}{2}VK(\mathbf{q})\begin{pmatrix}\begin{matrix}V\left|\psi_0\right|^4\big(K(\mathbf{q})[1-K(\mathbf{0})V\left|\psi_0\right|^2 L_1] \\ \times[J(\mathbf{q})+J^*(-\mathbf{q})] \\ -K(\mathbf{0})([K(\mathbf{q})V^2\left|\psi_0\right|^4-2]L_2^* \\ +K(\mathbf{q})J(\mathbf{q})J^*(-\mathbf{q})L_2\end{matrix} & \begin{matrix}\psi_0^2\big(-K(\mathbf{0})V\left|\psi_0\right|^2(K(\mathbf{q})V\left|\psi_0\right|^2 L_2 \\ \times[J(\mathbf{q})+J^*(-\mathbf{q})]+K(\mathbf{q})J(\mathbf{0})J(\mathbf{q})J^*(-\mathbf{q}) \\ +K(\mathbf{q})V^3\left|\psi_0\right|^6-2V\left|\psi_0\right|^2) \\ +K(\mathbf{q})J(\mathbf{q})J^*(-\mathbf{q})\big)\end{matrix} \\ \begin{matrix}\bar{\psi}_0^2\big(-K(\mathbf{0})V\left|\psi_0\right|^2(K(\mathbf{q})V\left|\psi_0\right|^2 L_2^* \\ \times[J(\mathbf{q})+J^*(-\mathbf{q})]+K(\mathbf{q})J^*(\mathbf{0})V^2\left|\psi_0\right|^4 \\ +K(\mathbf{q})J(\mathbf{q})J^*(-\mathbf{q})V\left|\psi_0\right|^2-2J^*(\mathbf{0})) \\ +K(\mathbf{q})V^2\left|\psi_0\right|^4\big)\end{matrix} & \begin{matrix}V\left|\psi_0\right|^4\big(K(\mathbf{q})[1-K(\mathbf{0})V\left|\psi_0\right|^2 L_1] \\ \times[J(\mathbf{q})+J^*(-\mathbf{q})] \\ -K(\mathbf{0})([K(\mathbf{q})V^2\left|\psi_0\right|^4-2]L_2^* \\ +K(\mathbf{q})J(\mathbf{q})J^*(-\mathbf{q})L_2\end{matrix}\end{pmatrix}, \tag{13}$$

$$
\mathcal{C}^{\mathcal{M}}_{-,+}(\mathbf{q}) = -\frac{1}{2}VK(\mathbf{q})
\begin{pmatrix}
\begin{array}{c}
V\left|\psi_0\right|^4 \big(K(\mathbf{q})[1-K(\mathbf{0})V\left|\psi_0\right|^2 L_1] \\
\times[J(\mathbf{q})+J^*(-\mathbf{q})] \\
-K(\mathbf{0})([K(\mathbf{q})V^2\left|\psi_0\right|^4-2]L_2 \\
+K(\mathbf{q})J(\mathbf{q})J^*(-\mathbf{q})L_2^*
\end{array}
&
\begin{array}{c}
\psi_0^2\big(-K(\mathbf{0})V\left|\psi_0\right|^2 (K(\mathbf{q})V\left|\psi_0\right|^2 L_2 \\
\times[J(\mathbf{q})+J^*(-\mathbf{q})]+K(\mathbf{q})J(\mathbf{0})V^2\left|\psi_0\right|^4 \\
+K(\mathbf{q})J(\mathbf{q})J^*(-\mathbf{q})V\left|\psi_0\right|^2-2J(\mathbf{0})) \\
+K(\mathbf{q})V^2\left|\psi_0\right|^4\big)
\end{array}
\\
\begin{array}{c}
\bar{\psi}_0^2\big(-K(\mathbf{0})V\left|\psi_0\right|^2 (K(\mathbf{q})V\left|\psi_0\right|^2 L_2^* \\
\times[J(\mathbf{q})+J^*(-\mathbf{q})]+K(\mathbf{q})J^*(\mathbf{0})J(\mathbf{q})J^*(-\mathbf{q} \\
+K(\mathbf{q})V^3\left|\psi_0\right|^6-2V\left|\psi_0\right|^2) \\
+K(\mathbf{q})J(\mathbf{q})J^*(-\mathbf{q})\big)
\end{array}
&
\begin{array}{c}
V\left|\psi_0\right|^4 \big(K(\mathbf{q})[1-K(\mathbf{0})V\left|\psi_0\right|^2 L_1] \\
\times[J(\mathbf{q})+J^*(-\mathbf{q})] \\
-K(\mathbf{0})([K(\mathbf{q})V^2\left|\psi_0\right|^4-2]L_2 \\
+K(\mathbf{q})J(\mathbf{q})J^*(-\mathbf{q})L_2^*
\end{array}
\end{pmatrix},
\tag{14}
$$

$$
\mathcal{C}^{\mathcal{M}}_{-,-}(\mathbf{q}) = -\frac{1}{2}VK(\mathbf{q})
\begin{pmatrix}
\begin{array}{c}
\left|\psi_0\right|^2 \big(K(\mathbf{q})[1-K(\mathbf{0})V\left|\psi_0\right|^2 L_1] \\
\times[J(\mathbf{q})^2+V^2\left|\psi_0\right|^4] \\
-K(\mathbf{0})J(\mathbf{q})L_1[K(\mathbf{q})V^2\left|\psi_0\right|^4-1]\big)
\end{array}
&
\begin{array}{c}
\psi_0^2\big(-K(\mathbf{0})K(\mathbf{q})V\left|\psi_0\right|^2 L_2 \\
\times[J(\mathbf{q})^2+V^2\left|\psi_0\right|^4] \\
+J(\mathbf{q})(K(\mathbf{q})V\left|\psi_0\right|^2 \\
-K(\mathbf{0})L_2[K(\mathbf{q})V^2\left|\psi_0\right|^4-1])\big)
\end{array}
\\
\begin{array}{c}
\bar{\psi}_0^2\big(-K(\mathbf{0})K(\mathbf{q})V\left|\psi_0\right|^2 L_2^* \\
\times[J(\mathbf{q})^2+V^2\left|\psi_0\right|^4] \\
+J(\mathbf{q})(K(\mathbf{q})V\left|\psi_0\right|^2 \\
-K(\mathbf{0})L_2^*[K(\mathbf{q})V^2\left|\psi_0\right|^4-1])\big)
\end{array}
&
\begin{array}{c}
\left|\psi_0\right|^2 \big(K(\mathbf{q})[1-K(\mathbf{0})V\left|\psi_0\right|^2 L_1] \\
\times[J(\mathbf{q})^2+V^2\left|\psi_0\right|^4] \\
-K(\mathbf{0})J(\mathbf{q})L_1[K(\mathbf{q})V^2\left|\psi_0\right|^4-1]\big)
\end{array}
\end{pmatrix}.
\tag{15}
$$
